# Tribute of Respect—Dr. M. J. Ely

**Published:** 1883-10

**Authors:** W. D. Bizzell, K. C. Divine, E. L. Connally, W. S. Elkin

**Affiliations:** Committee; Atlanta, Ga.; Committee; Atlanta, Ga.; Committee; Atlanta, Ga.; Committee; Atlanta, Ga.


ATLANTA, GA., Oct. 8, 1883.

AT a called meeting of the Atlanta Medical Society, the committee reported the following resolutions, which were unanimously adopted:

*Gentlemen of the Atlanta Medical Society:*

Your Committee beg to report the following resolutions on the death of Dr. M. J. Eley:

It is with profound regret that the Atlanta Medical Society learns of the death of Dr. M. J. Eley. Though only a few months resident of Atlanta, Dr. Eley, by his gentlemanly bearing, urbanity and professional attainments, had won our esteem and confidence; and his death is a sad loss to the community and the profession.

That we tender to his bereaved family our sincere condolence in their deep affliction.

We recommend that the President appoint a committee of four to escort his remains to the train which will bear him to his old home in Alabama.

W. D. BIZZELL, M.D.,
K. C. DIVINE, M.D.,
E. L. CONNALLY, M.D.,
W. S. ELKIN, M.D.,
Committee.

